# Patterns of undertreatment among patients with acute myeloid leukemia (AML): considerations for patients eligible for non-intensive chemotherapy (NIC)

**DOI:** 10.1007/s00432-021-03756-7

**Published:** 2021-08-30

**Authors:** Elizabeth Hubscher, Slaven Sikirica, Timothy Bell, Andrew Brown, Verna Welch, Alexander Russell-Smith, Paul D’Amico

**Affiliations:** 1 Purple Squirrel Economics, a Cytel Company, Waltham, MA USA; 2 Pfizer Inc., New York, NY USA

**Keywords:** Acute myeloid leukemia, Treatment patterns, Undertreatment, Non-intensive chemotherapy, Real-world evidence

## Abstract

Acute myeloid leukemia (AML) is a life-threatening malignancy that is more prevalent in the elderly. Because the patient population is heterogenous and advanced in age, choosing the optimal therapy can be challenging. There is strong evidence supporting antileukemic therapy, including standard intensive induction chemotherapy (IC) and non-intensive chemotherapy (NIC), for older patients with AML, and guidelines recommend treatment selection based on a patient’s individual and disease characteristics as opposed to age alone. Nonetheless, historic evidence indicates that a high proportion of patients who may be candidates for NIC receive no active antileukemic treatment (NAAT), instead receiving only best supportive care (BSC). We conducted a focused literature review to assess current real-world patterns of undertreatment in AML. From a total of 25 identified studies reporting the proportion of patients with AML receiving NAAT, the proportion of patients treated with NAAT varied widely, ranging from 10 to 61.4% in the US and 24.1 to 35% in Europe. Characteristics associated with receipt of NAAT included clinical factors such as age, poor performance status, comorbidities, and uncontrolled concomitant conditions, as well as sociodemographic factors such as female sex, unmarried status, and lower income. Survival was diminished among patients receiving NAAT, with reported median overall survival values ranging from 1.2 to 4.8 months compared to 5 to 14.4 months with NIC. These findings suggest a proportion of patients who are candidates for NIC receive NAAT, potentially forfeiting the survival benefit of active antileukemic treatment.

## Introduction

AML is a life-threatening hematologic malignancy most common in older adults (SEER 2020). In the US alone, 19,940 new cases of AML and 11,180 deaths were projected for 2020 (SEER 2020). The prognosis of AML is bleak, particularly for patients aged 65 years and older who represent 58.9% of new cases in the US but account for 72.5% of AML-related deaths. In this population, 1-year survival rates are ≤ 30% (Dores et al. 2012; Ocias et al. 2016; Osca-Gelis et al. 2015; Shah and Ghimire 2014; Thein et al. 2013).

The poor prognosis of older patients with AML is a function of a complex set of biological and clinical factors. The population is highly heterogenous, exhibiting wide variability in health history, performance and functional status, comorbidities, organ dysfunction, polypharmacy, cytogenetic abnormalities, and social support that may influence the ability to tolerate treatment, resistance to available therapies, and likelihood of receiving optimal treatment (Eleni et al. 2010; Wheatley et al. 2009). Other disease factors, such as etiology (de novo, secondary, or therapy-related AML [t-AML]) and baseline bone marrow blasts also may impact a patient’s disease course, treatment, and outcomes (DiNardo et al. 2016; Stone et al. 2019).

Newly diagnosed patients with AML may be treated with IC, followed by hematopoietic stem cell transplant (HSCT) and/or consolidation therapy. Lower-intensity treatments such as hypomethylating agents (HMAs) have been available to patients who are not candidates for IC since the mid-2000s. Newer non-intensive treatments such as glasdegib and venetoclax, as well as targeted treatments, including fms-like tyrosine kinase 3 (FLT3) and isocitrate dehydrogenase (IDH) inhibitors, and the antibody–drug conjugate gemtuzumab ozogamicin, became available after 2016 (Hitzler and Estey 2019; Lancet 2018; Wang 2014). All patients receive some form of BSC, which includes some combination of infection control, pain relief, transfusions, and antiemesis.

Determining candidacy for IC or NIC is not standardized. Existing international treatment guidelines recommend against using age as the only determining factor, but no consensus algorithm has been published to date (Döhner et al. 2017; Tallman et al. 2019). In addition to age, comorbidities, performance status (PS), and disease characteristics, physicians account for patient factors including treatment preference, financial and other resources, and available support systems (Kantarjian et al. 2010). Evidence to aid decision-making is often conflicting and limited, in part because clinical trials generally exclude patients on the basis of poor PS, confounding comorbidities, and advanced age (Kantarjian et al. 2010; Medeiros et al. 2018). Both providers and patients are tasked with balancing the risks of adverse events and treatment-related mortality and benefits of anti-cancer therapy (intensive or non-intensive) for older patients with AML (Wang 2014).

With a lack of consensus and complex considerations, the optimal treatment of older patients with AML presents a distinct challenge for physicians. Unsurprisingly, wide variation in treatment selection exists (Loberiza et al. 2014); moreover, analyses of real-world data regarding therapeutic utilization in AML suggest that a substantial number of patients receive NAAT despite published evidence that AML patients, including those over age 65, benefit from receiving active antileukemia treatment (Menzin et al. 2002; Oran and Weisdorf 2012).

We sought to examine real-world treatment patterns in AML, including factors associated with the selection of NAAT as opposed to NIC or IC and survival outcomes to provide insight into the factors that influence treatment selection and the impact of undertreatment, or receipt of NAAT by a patient who may benefit from NIC treatment.

## Methods

We conducted a focused literature review that concentrated on rates of undertreatment, as well as patient characteristics and survival outcomes, using an iterative hybrid of pearl growing and snowball search methods (LibGuides 2020; Ramer 2005; Zwakman et al. 2018). Briefly, an initial broad search was conducted to identify a set of core publications. The abstracts of initial records were screened and core publications were selected based on the relevance of patient population, interventions, and outcomes to the research question as well as large sample sizes that were representative of the heterogenous AML patient population. These core publications were then used for prospective searches (e.g., cited by) and retrospective bibliographic searches, as well as the identification of new index terms for additional searches.

The initial search was conducted in PubMed and employed the following search terms: “treatment pattern”, “treatment decision” and “treatment utilization” and the MeSH heading “Acute Myeloid Leukemia”. Search limitations were defined as English language articles only, dated between January 2010 and the date of the initial search (May 2020).

## Results

 A total of 831 initial abstract records were identified, from which nine core publications were selected and used for additional prospective, retrospective, and index-term searches. Overall, 86 records that described the proportion of patients receiving either IC, NIC, or NAAT were identified. Among these, 25 reported the proportion of patients who received NAAT. Most of the identified studies were retrospective analyses of large databases, while some analyzed registries and multicenter data. Eleven studies analyzed single-center data or were prospective. Several regions were represented, including the US, EU, UK, Asia, Scandinavia, Israel, and Brazil. Forty-seven studies limited populations to patients > 60 years or had a median age of ≥ 60 years. Among 24 studies reporting median patient age, the pooled average was 70.6 years (range 36–78).

### Rates of no active antileukemic treatment

Among the 25 studies reporting the proportion of patients receiving NAAT, the majority (57%) were retrospective observational studies. Ten were single-center, while the rest were multicenter or analyzed registries or large databases, such as Programa para el Tratamiento de Hemopatias Malignas (PETHEMA), California Cancer Registry, National Cancer Database (NCDB), Surveillance Epidemiology and End Results Program (SEER) database, Centers for Medicare and Medicaid Services’ claims, or commercially available claims databases. The payer databases provided data for both inpatient and outpatient medication claims. One study was prospective (Berger et al. 2019). The number of included patients varied widely, from 61 in a single-center study to 98,293 in an analysis of the NCDB. Overall, the identified studies represented a total of 220,569 patients with AML from regions including the US, EU, UK, Japan, Brazil, India, Israel, and Serbia.

In the US, the percentage of patients who received NAAT ranged from 10 to 61.4%, with a weighted average of 30%, whereas 24.1–35% of patients in the EU received NAAT (Table 1). Rates of active treatment in the rest of the world varied widely. In India, 65.5% of patients received NAAT (Kanakasetty et al. 2019). According to a small single-center study by Neaman et al. in 2019, 15.6% of Israeli patients with AML received NAAT (Neaman 2020).

**Table 1:** Reported rates of NAAT in identified studies

Study reference	Country	NAAT (%)	Study population (*N*)	Date range
Talati (2020)	US	28	980	1995–2016
Bhatt (2018)	US	25.3	61,775	2003–2011
Ma (2016)	US	43	1139	2005–2015
Medeiros (2015)	US	60	8336	2000–2009
Meyers (2013)	US	57	4058	1997–2007
Tu (2019)	US	18	2879	2012–2018
Willner (2019)	US	26.2	61	2000–2017
Zeidan (2019)	US	52.7	14,089	2001–2013
Oran (2012)	US	61.4	5480	2000–2007
Percival (2019)	US	10	442	2014–2016
Goyal (2015)	US	24	98,293	2003–2013
Hagiwara (2018)	US	32	9455	2007–2016
Solomon (2020)	US	19	323	2009–2017
Berger (2019)	France	30.2	592	2009–2014
Deschler (2013)	Germany	24.1	195	2004–2008
Heiblig (2017)	France	35	302	2000–2014
Acuna-Cruz (2020)	Spain	28	2637	1999–2013
Martinez-Cuadrón (2020)	Spain and Portugal	25	8521	1990–2019
Yanada (2015)	Japan	27	158	2004–2012
Serna (2020)*	Spain	29.6	135	2009–2019
Colovic (2012)	Serbia	34.8	210	2001–2006
Sandes (2011)	Brazil	51.6	31	2003–2008
Neaman (2020)	Israel	50; 15.6	44; 32	2017–2018; 2019
Kanaksetty (2019)	India	65.5	402	2013–2017

*N* number; *NAAT* no active antileukemia treatment

*This study population included only patients who were not candidates for IC

#### Temporal trends

In identified studies, rates of NAAT among patients with AML show a downward trend in the last two decades (Hagiwara et al. 2018; Medeiros et al. 2015; Meyers et al. 2013; Zeidan et al. 2019). According to an analysis of the SEER-Medicare database, patients diagnosed in 2000 or later were more likely to receive antileukemia treatment than patients diagnosed between 1997 and 1999: the odds ratio (OR) was 1.139 for those diagnosed from 2000 to 2003 and 1.310 for those diagnosed from 2004 to 2007 (Meyers et al. 2013). This effect was durable in more recent years. In an analysis of the NCDB, patients diagnosed with AML after the reference period of 2003–2006 were more likely to receive antileukemia treatment; for those diagnosed from 2007 to 2010, the OR was 1.11 (95% CI 1.08–1.15), and for those diagnosed from 2011 to 2013, the OR was 1.27 (95% CI 1.22–1.31) (Goyal et al. 2015).

**Fig. 1:**
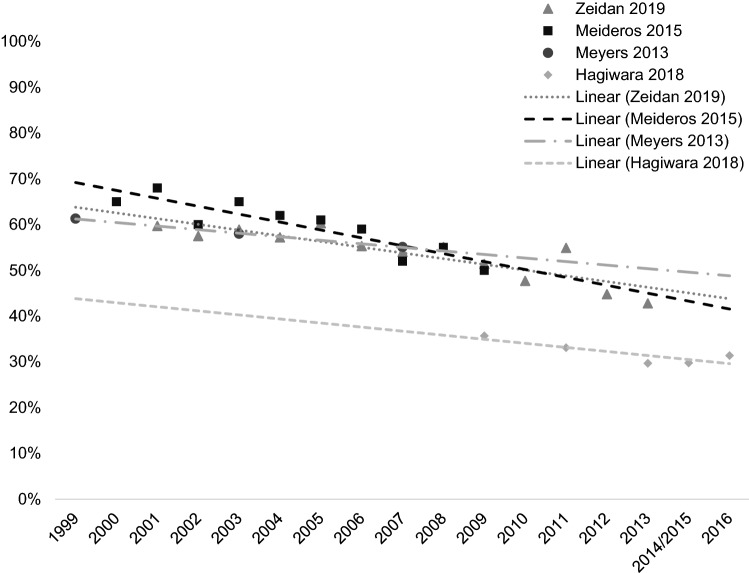
Rates of NAAT over time

Four studies provided some annual rates of NAAT, which were scatter plotted, and trend lines (dashed lines) were added subsequently (Fig. 1).

### Factors associated with treatment selection

#### Patient characteristics

Twelve studies analyzed the characteristics of patients in either the antileukemia treatment or NAAT groups. Patients who received NAAT tended to be older, with 12 studies reporting an association between increased age and decreased likelihood of receiving antileukemia treatment. The majority of studies reported patient comorbidity burden and/or PS. The Charlson Comorbidity Index was the most commonly used assessment (nine studies), followed by National Cancer Institute Comorbidity Index (three studies), HCT-CI (two studies), and Elixhauser comorbidity score (one study). Overall, a higher comorbidity index or presence of a chronic comorbid condition that was not optimally controlled was the second most common factor associated with receipt of NAAT: nine of 13 studies reported an association (Table 2). The most frequently reported comorbidities were cardiovascular disease and diabetes. Chronic kidney disease, chronic obstructive pulmonary disease, and dementia were also associated with a higher probability of receiving NAAT compared to any active treatment (Hagiwara et al. 2018). Though few studies provided data regarding treatment decision criteria, the presence of an active suboptimally managed medical condition, severe cardiovascular disease, or diabetes and cardiovascular disease were provided as reasons for selecting NAAT (Colovic et al. 2012; Serna 2020). Either advanced age or the presence of a comorbidity was cited as the reason for choosing not to treat a patient with chemotherapy in 11.4% of cases in the NCDB (Medeiros et al. 2015).

**Table 2:** Clinical characteristics associated with NAAT status in identified studies

Study reference	Age	Comorbidity burden	PS
Oran (2012)	✓ ≥ 70 years	✓ CCI ≥ 1	–
Meyers (2013)	✓ ≥ 75 years	✓ CCI ≥ 1	–
Medeiros (2015)	✓ NAAT patients were significantly older (81 vs 75 years, *p* < 0.0001)	✓ CCI ≥ 1	✓ PPI
Goyal (2015)	✓ Elderly	✓ ≥ 1 comorbid condition	–
Patel (2015)	✓ Increasing age*	✓ CCI ≥ 1	–
Yanada (2015)	✓ ≥ 75 years	–	✓ ECOG PS 3–4
Hirsch (2015)	✓ NAAT patients were significantly older (79 vs 77 years, *p* = 0.0082)	–	✓ ECOG PS ≥ 2
Heiblig (2017)	–	–	✓ ECOG PS > 2
Hagiwara (2018)	✓ ≥ 60 years	✓ Higher mean CCI; history of COPD, CKD, dementia, diabetes	–
Bhatt (2018)	✓ ≥ 60 years	✓ Higher	–
Zeidan (2019)	✓ ≥ 70 years	✓ ≥ 3 comorbidities	–
Martinez (2020)	✓ Increasing age*	–	✓ Worse ECOG PS
Acuna-Cruz (2020)	✓ NAAT patients were significantly older (79 vs 76 years, *p* < 0.001)	–	✓ ECOG 3–4 PS

✓ Associated with receipt of NAAT, followed by study definition of characteristic; – not assessed

*CCI* charlson comorbidity index; *CKD* chronic kidney disease; *COPD* chronic obstructive pulmonary disease; *ECOG PS* Eastern Cooperative Oncology Group performance status; *NAAT* no active antileukemia treatment; *PPI* poor performance indicators

*Study observed a significant difference in NAAT for all patient groups above youngest group (18–29 and 18–21 years); however, the effect was more marked in patients with increasing age and > 60 years

With respect to PS, the Eastern Cooperative Oncology Group (ECOG) PS was the most frequently used (eight studies). In five studies, the proportion of patients with poor ECOG PS (ranging from ≥ 2 to 3–4) was higher among the group that received NAAT compared to those patients receiving chemotherapy or targeted treatment. Poor PS (ECOG PS 4) was a provided reason for the selection of NAAT in one study in Serbia (Colovic et al. 2012). Because the SEER database does not capture PS, Medeiros et al. assessed poor performance indicators (PPI) such as supplemental oxygen, wheelchair, home health, and skilled nursing claims. The presence of PPI in the year prior to AML diagnosis was significantly higher among the population receiving NAAT (Medeiros et al. 2015). A higher number of prior outpatient pharmacy claims was also observed in the patient population NAAT (Hagiwara et al. 2018).

#### Disease characteristics

Several of the identified studies characterized disease characteristics in both the antileukemia treatment and NAAT populations (Table 3). In two studies, rates of secondary AML were higher in the NAAT population (Oran and Weisdorf 2012; Medeiros et al. 2015). Patients with a prior history of myelodysplastic syndrome (MDS) or with t-AML were less likely to receive antileukemia treatment. History of previous MDS was a criteria for selecting NAAT among patients who did not have poor PS in two studies (Colovic et al. 2012; Sandes et al. 2011). Of note, an analysis of the PharMetrics Plus-IMS Hospital Charge Detail Master database demonstrated a positive association between the history of MDS and receipt of chemotherapy (Hagiwara et al. 2018). While the rate of NAAT was 32% in the total AML population, it was 28% among patients with t-AML or AML with myelodysplastic-related changes.

**Table 3:** Disease-related characteristics associated with NAAT status

Study reference	Type of AML	Cytogenetic risk category	Other hematologic factor
Oran (2012)	Secondary AML	–	–
Medeiros (2015)	Secondary AML	–	–
Hirsch (2015)	–	Unfavorable risk	–
Hagiwara (2018)	No*	–	–
Martinez (2020)	–	Adverse	Higher baseline WBC and BMB

*AML* acute myeloid leukemia; *BMB* bone marrow blast; *WBC* white blood cell

*Secondary AML was positively associated with receipt of antileukemia treatment in this study

– not assessed

Patients with adverse cytogenetic risk were also more likely to receive NAAT in studies including patients with AML in the US, France, Spain, and Portugal (Bhatt et al. 2018; Hirsch et al. 2015; Martinez-Cuadron 2020). Unfavorable cytogenetics were reported criteria for receiving NAAT according to a treatment algorithm for elderly patients in an academic hospital in Brazil (Sandes et al. 2011). Other factors reported in single studies were higher baseline bone marrow blasts and white blood cell counts (Martinez-Cuadron 2020).

#### Sociodemographic factors

In eight identified studies, sociodemographic factors were associated with a type of treatment (Table 4). Females were significantly less likely than males to receive antileukemia treatment in seven studies representing patients from the SEER-Medicare, NCDB, California Cancer Registry, and PharMetrics databases with index dates ranging from 1998 through 2016 (Hagiwara et al. 2018; Medeiros et al. 2015; Zeidan et al. 2019; Goyal et al. 2015; Bhatt et al. 2018; Patel et al. 2015). In an analysis of the SEER database from 1997 to 2007, females had an OR of receipt of antileukemia treatment of 0.944 compared to males; however, this was not statistically significant (Meyers et al. 2013).

**Table 4:** Sociodemographic characteristics associated with NAAT in identified studies

Reference	Sex	Race/ethnicity	Marital status	Economic status^a^	Education	Treatment setting
Medeiros (2015)	Female	No	Unmarried	Lower income	–	–
Oran (2012)	Female	No	–	Lower income	–	–
Goyal (2015)	Female	No**	–	Lower income	–	Community hospital
Hagiwara (2018)	Female	–	–	–	–	–
Meyers (2013)	No	Black	–	–	–	–
Patel (2015)	Female	Black	–	–	–	–
Bhatt (2018)	Female	Black	–	Lower income, less insured	Lower educational status	Non-academic center, lower volume
Zeidan (2019)	Female*	No	Unmarried	Lowest income quartile	–	–

*Except age 66–69, where men were more likely to receive NAAT

**Black patients had increased odds of receiving active treatment compared to White patients (OR 1.20; 1.13–1.26)

^a^Income was defined based on median household income by zipcode and assessed in quartiles (0–25th%, 26th–50th%, 51st–75th%, 76th–100th%); actual numeric income ranges varied depending on year of data collection

– not assessed; No: association was not observed

The effect of race on treatment selection was unclear. In three studies including patients from the PharMetrics database, the California Cancer Registry, and the NCDB, Black race was associated with lower odds of receiving antileukemia treatment (OR range 0.67–0.85) (Meyers et al. 2013; Bhatt et al. 2018; Patel et al. 2015); however, this association was not observed in separate analyses of the SEER-Medicare database covering patients diagnosed between 2000 and 2013 (Oran and Weisdorf 2012; Medeiros et al. 2015; Zeidan et al. 2019). One analysis of the NCDB from 2003 to 2013 revealed higher odds of systemic treatment for Black patients with AML compared to White patients (OR 1.20; 1.13–1.26) (Goyal et al. 2015).

Other identified factors associated with not receiving antileukemia treatment included lower household income status (five studies) and unmarried status (two studies). Patients who were treated at academic centers and/or hospitals with higher volume, travelled longer distances to receive treatment, reported a visit with an oncologist or hematologist within 1 year of AML diagnosis, or received the influenza vaccine were more likely to receive antileukemia treatment (Goyal et al. 2015; Bhatt et al. 2018). Patients with a diagnosed mental disorder or disability had an increased odds of receiving NAAT (OR 1.43 and 2.31, respectively) (Zeidan et al. 2019).

#### Patient preference

Though the majority of cases not receiving chemotherapy in the NCDB did not specify a reason, 11.3% cited patient or family refusal (Bhatt et al. 2018). In a study of patients in India between 2014 and 2017, 53.3% of patients refused active treatment for a variety of reasons including insufficient or absent family support, travel/relocation constraints, and financial limitations (Kanakasetty et al. 2019). In a discrete choice experiment, two preference patterns emerged for either short-term side effect avoidance or complete remission achievement (Richardson et al. 2020). Women and patients over the age of 60 years were significantly more likely to be side-effect avoidant, whereas patients with private insurance were more likely to seek remission. Interestingly, a survey of patients and practitioners revealed a discordance between patients’ recollection of discussions with healthcare providers regarding treatment options. Furthermore, 21% of surveyed patients reported discussing BSC alone and 28% reported that the treatment decision-making process did not match their preference (El-Jawahri et al. 2019).

#### Outcomes with treatment

Overall, patients who received NAAT in the identified studies had worse overall survival (OS) than those who received active antileukemia treatment, with reported median OS ranging from 1.2 to 4.8 months vs 5 to 14.4 months, respectively (Table 5). Across these heterogeneous patient populations, the weighted average OS among patients receiving NAAT was 1.7 months, whereas it was 8.0 months among patients receiving NIC. In two studies, patients who received treatment had 45–65% reduction in risk of death compared with patients who received NAAT (Medeiros et al. 2015; Willner et al. 2019).

**Table 5:** Survival outcomes with NAAT and NIC

Study reference	mOS with NAAT (months)	mOS with NIC (months)
Talati (2020)	2.1	14.4
Ma (2016)	4.8	8.6
Medeiros (2015)	1.5	5.0
Willner (2019)	2.0	10.0
Martinez (2020)	1.2	9.0
Acuna-Cruz (2020)	1.2	7.8
Heiblig (2017)	2.6	11.5

*mOS* median overall survival; *NAAT* no active antileukemia treatment; *NIC* non-intensive treatment

## Discussion

Despite an observable trend toward increased use of antileukemia treatment among patients with AML in recent years, there remains a substantial portion of patients who do not receive therapy beyond BSC. In our review, the most common factor associated with receipt of NAAT was increasing age. Current AML treatment guidelines recommend that age should not be the sole determinant of a patient’s eligibility for treatment, however, the pervasiveness of this finding suggests physicians may continue to strongly consider age in treatment decisions. Survival gains for younger patients with AML in recent decades have not been observed to the same degree in older patients (SEER 2020; Thein et al. 2013; Shallis et al. 2019). This disparity may be due, at least in part, to undertreatment. Older age is inherently linked to other important characteristics that may influence a patient’s ability to tolerate and benefit from treatment, including frailty, presence of comorbidities, decreased organ function, and diminished PS. Frailty is not universally measured and was not reported in the studies that we identified; however, other characteristics such as ECOG PS and comorbidities were analyzed in univariate analyses in a number of the studies, with mixed results. Of note, among studies reporting a relationship between older age and receipt of NAAT, poor PS or elevated comorbidity burden were also independently linked to receipt of NAAT. Reported ORs showed a more dramatic impact with age than comorbidity burden; however, the observed age effect may have been attenuated if adjustments for comorbidity burden had been performed (Oran and Weisdorf 2012; Meyers et al. 2013; Goyal et al. 2015; Patel et al. 2015). Bhatt et al. (2018) was the only identified study that reported results of multivariable modeling examining the interaction of age and comorbidity burden on the likelihood of receiving NAAT (Bhatt et al. 2018). Among patients over age 41 years, elevated risk of receiving NAAT with increasing age holds and was consistent across patient groups with increasing Charlson Comorbidity Index. Future analyses of multivariable models will help to further elucidate the role of age alone as well as the contribution of comorbidities and other important characteristics including frailty and PS on treatment decisions and patients’ ability to tolerate and benefit from treatment.

It is possible that some physicians employ criteria intended to determine a patient’s candidacy for IC to exclude them from NIC as well. According to a survey of 100 hematologists, 5% expressed a preference for using BSC only to treat patients who were either elderly or not candidates for IC (Portugal and Nucci 2019). Percival et al. (2019) reported the lowest rate of NAAT (10%). This study was unique in that researchers examined patients aged 75 years or older who met AML clinical trial inclusion criteria of PS 0–2, glomerular filtration rate at or below 60 ml/min, alanine transaminase at or below twice the upper limit of normal, bilirubin ≤ 1.5 mg/dl, and measures of cardiac health including no history of myocardial infarction, congestive heart failure, or left ventricular ejection fraction below 49%, suggesting that clinicians were more likely to treat patients that met IC treatment criteria (Percival et al. 2019). In identified studies, clinical factors such as poor PS and the presence of comorbidities were associated with NAAT. Two studies specified poor PS as a criterion for receiving NAAT (Colovic et al. 2012; Sandes et al. 2011). Concomitant conditions such as cardiovascular disease, diabetes, and chronic kidney disease were reported as decision drivers for NAAT (Colovic et al. 2012; Bhatt et al. 2018). While such biologic factors may confer a poorer prognosis, in some cases these patients benefit from treatment. In a clinical trial of glasdegib, nearly 67% of patients with ECOG PS 2 treated with glasdegib plus low-dose cytarabine achieved complete remission (Cortes et al. 2020). Additionally, trials of both glasdegib and venetoclax allowed patients with cardiovascular disease and post-hoc analyses of extended follow-up data demonstrated survival benefits over low-dose cytarabine alone (Cortes et al. 2020; Wei et al. 2020).

Our review also identified reports of other non-clinical factors that were more common among patients who received NAAT. Several US studies demonstrated an association between female sex, lower household income, and unmarried status and the receipt of NAAT (Oran and Weisdorf 2012; Hagiwara et al. 2018; Medeiros et al. 2015; Meyers et al. 2013; Zeidan et al. 2019; Goyal et al. 2015; Bhatt et al. 2018). Though none of the identified studies established a correlation between these patient characteristics and the reported treatment decisions, such patterns warrant further exploration. An association between Black race and receipt of NAAT was reported in three studies; however, this association was not observed in the SEER-Medicare database and in one of two analyses of the NCDB. Though evidence is mixed, sociodemographic factors do appear to influence survival outcomes. There is evidence that the risk of death among patients with AML is higher in those who live alone and are not married as well as among patients with lower income levels (Costa et al. 2016). Black Americans with AML also have worse survival than other races (Byrne et al. 2011; Pulte et al. 2013).

The current literature review identified studies comprising a large population of patients diagnosed with AML over nearly three decades, from 1990 to 2019. Analyzed data represented a wide range of regions and were derived from several different real-world evidence sources, including payer databases, registries, and single- and multicenter data. While the identified studies examined treatment patterns, characteristics of patients receiving types of therapy, and factors involved in decision making as well as overall patient outcomes, there was a great deal of variability in the data reporting, both in terms of analyses and the available input data depending on the source. In terms of our search strategy, though the pearl growing/snowball method is designed to provide comprehensive retrieval, the selection of core publications creates the possibility of selection bias, and ultimately the review is limited by the quality and quantity of retrieved studies and data. The majority of identified studies were retrospective observational analyses not designed to elucidate reasons for treatment selection. Moreover, it is impossible to discern whether worse outcomes among patients with NAAT are a result of undertreatment or if these patients were treated supportively because they had poor prognoses at the time of diagnosis. While non-clinical factors appear to play a role in driving treatment decisions, our assessment of the data is descriptive and did not ascertain the influence of certain factors in terms of treatment selection or outcomes. Longitudinal analyses of individual patient data would provide more robust and comprehensive insights.

Since 2017, new NIC options beyond low-dose cytarabine and HMAs have been available to extend survival in patients with AML who are not candidates for IC; however, the findings of this review suggest a substantial proportion of patients who might benefit from such treatment do not receive it (Wang 2014; Shallis et al. 2019; Bories et al. 2018; Griffiths et al. 2020; Palmieri et al. 2020). Across identified studies, patients treated with NIC experienced longer OS than those who received NAAT. Based on the proportion of patients in the identified studies who received NAAT instead, this may represent a potentially substantial amount of life years lost to undertreatment. It is important to note that most of the treatment and outcome data in this review were based on timeframes before these treatments were widely available; thus, practice shifts based on recent NIC approvals may not be reflected. Regardless, treatment decisions for patients with AML remain challenging and must consider the patient’s overall situation. Certain non-clinical factors appear to negatively impact a patient’s likelihood of receiving antileukemia treatment. Enhanced understanding of key considerations influencing treatment selection and survival outcomes will aid in the development of consensus guidelines to guide and inform complex clinical decisions. Furthermore, improved access and support for patients in more vulnerable populations will create better opportunities for parity and optimal care.

## Data Availability

All data is provided in the manuscript.
